# Effects of Increased Vigilance for Locomotion Disorders on Lameness and Production in Dairy Cows 

**DOI:** 10.3390/ani3030951

**Published:** 2013-09-13

**Authors:** Yasmin Gundelach, Timo Schulz, Maren Feldmann, Martina Hoedemaker

**Affiliations:** Clinic for Cattle, University of Veterinary Medicine Hannover, Bischofsholer Damm 15, 30173 Hannover, Germany; E-Mails: timo.schulz@tiho-hannover.de (T.S.); maren.feldmann@tiho-hannover.de (M.F.); martina.hoedemaker@tiho-hannover.de (M.H.)

**Keywords:** locomotion score, dairy cow, early treatment, lameness, lameness duration

## Abstract

**Simple Summary:**

For animal welfare reasons, reducing the prevalence of lameness should be one of the most important goals in dairy farming. In this study, the influence of early detection and treatment of lame cows on lameness prevalence, incidence and duration of lameness in comparison with routine lameness management practiced on a dairy farm was determined. The results suggest that early detection and treatment of lame cows significantly reduced the duration of lameness, and, therefore, the prevalence of lameness.

**Abstract:**

The objective of this study was to determine the influence of weekly locomotion scoring and, thus, early detection and treatment of lame cows by a veterinarian on lameness prevalence, incidence, duration of lameness, fertility and milk yield on one dairy farm in Northern Germany. Cows were distributed to two groups. Cows in Group A (n = 99) with a locomotion score (LS) > 1 were examined and treated. In Group B (n = 99), it was solely in the hands of the farmer to detect lame cows and to decide which cows received treatment. Four weeks after the beginning of the experimental period, the prevalence of cows with LS = 1 was higher in Group A compared with Group B. Prevalence of lame cows (LS > 1) increased in Group B (47.6% in Week 2 to 84.0% in Week 40) and decreased in Group A from Week 2 to Week 40 (50% to 14.4%; *P* < 0.05). Within groups, the monthly lameness incidence did not differ. The average duration of lameness for newly lame cows was 3.7 weeks in Group A and 10.4 weeks in Group B (*P* < 0.001). There was no effect on fertility and incidence of puerperal disorders. The 100-day milk yield was calculated from cows having their first four Dairy Herd Improvement (DHI) test day results during the experimental period**.** The mean 100-day milk yield tended to be higher in Group A compared with Group B (3,386 kg *vs.* 3,359 kg; *P* = 0.084).

## 1. Introduction

Lameness in cattle is considered to be an important health problem in dairy herds. Lameness has a negative impact on milk production [1,2,3], reproduction [4,5] and poses an increased risk of culling [4,6]. In addition to the economic impact, lameness is a behavioral expression of pain. Indeed, lameness is one of the most important welfare problems in dairy cattle [7]. In the study by Tadich *et al.* [8], the mean prevalence was 33.2% in large herds and 28.7% in small herds in southern Chile. Sogstad *et al.* [9] reported a prevalence of 22.6%.

Whay *et al.* [10] revealed that the farmer’s perception of the prevalence of lameness in a herd was lower than that of a researcher who observed all cows individually, looking for lameness. Leach *et al.* [11] also suggested that farmers were not fully aware of the numbers of lame cows in their herds. Based on a questionnaire carried out with 222 UK dairy farmers, Leach *et al.* [11] concluded that farmers underestimated the extent of lameness and the implications for the performance of their cows and their business. Leach *et al.* [12] tested an ‘early threshold’ protocol for treating cows within 48 h of being detected to be mildly or severely lame (mobility was scored at 14 day intervals). The early threshold schedule in their study resulted in drastically reducing the time to treatment and the early treatment also reduced the prevalence of lameness.

Green *et al.* [2] concluded that some of the potential of high yielding cows in a herd might be lost if they went lame. In their study, decreased milk yield occurred from 4 month before until 5 month after a cow was diagnosed as clinically lame. They underlined the importance of early identification of clinical lameness and the necessity for techniques to improve this highly subjective diagnosis. Early and effective treatment following immediate identification of lame cows will result in cost improvements at farm level [13]. 

In order to determine the impact of a strict lameness management program including weekly locomotion scoring and immediate treatment of all lame cows by a veterinarian, data on the prevalence, incidence and duration of lameness were collected on one dairy farm in Northern Germany known to have a high prevalence of lame cows. Additionally, an analysis of milk yield, animal health and fertility was performed. 

## 2. Experimental Section

### 2.1. Experimental Farm

A dairy farm with 144 lactating cows in Lower Saxony, Germany, served as the experimental farm. Cows were kept in a free stall barn with slatted floor. The cubicles had a raised concrete stall floor and were equipped with rubber mats covered with lime. The floor and cubicles were cleaned at irregular intervals either manually or mechanically using scrappers. Lactating cows, heifers and dry cows were kept in different pens. Approximately 14 days before calving, dry cows were transferred to a straw yard. Close to the calculated calving date they were brought to the straw maternity pens, which had space for two to three animals. The average annual herd milk yield was 8,978 kg.

### 2.2. Experimental Animals

All experimental animals belonged to “German Holstein breed”. At the beginning of the study all lactating and dry cows were matched regarding parity and days in milk and then allocated by stratified random sampling to two groups. Heifers were randomly allocated to one of the two groups after calving. 

### 2.3. Experimental Design

The experimental period was between March 2007 and December 2007 covering 41 weeks. Before and after this period, functional claw trimming was performed by a professional claw trimmer on all lactating and dry cows and all diagnoses were recorded. Heifers were integrated after calving and, therefore, only included in the second claw trimming date. Locomotion scoring of the whole lactating herd according to Sprecher *et al.* [14] was carried out weekly over a period of 41 weeks under the same conditions after morning milking in the walkway behind the milking parlor by a veterinarian using a five point scale (Table 1). Cows were visually identified by ear tag number. The cows in the two groups were not marked in a particular way. Thus, the group they belonged to was not apparent. The veterinarian who conducted the locomotion scoring was oblivious to the fact whether cows were in Group A or B. The first scoring date (Week 1) was immediately before the first herd claw trimming, the penultimate scoring date in Week 40 was before the second claw trimming. In Week 39, locomotion scores could not be determined due to an accident suffered by the study veterinarian. 

**Table 1:** Locomotion scores and descriptions developed by Sprecher *et al.* [14].

Locomotion score	Clinical description	Comments
1	Normal	Stands and walks normally. All feet placed with purpose.
2	Mildly lame	Stands with a flat back, but arches when walks; gait is slightly abnormal.
3	Moderately lame	Stands and walks with an arched back; short strides with one or more legs.
4	Lame	Arched back standing and walking; one or more limbs favored, but at least partially weight bearing.
5	Severely lame	Arched back; refuses to bear weight on one limb; may refuse or have great difficulty moving from lying position.

For a given week, cows in Group A with a locomotion score >1 were examined within the following 5 days by a skilled veterinarian using a hoof care chute and treated accordingly. All diagnoses were documented. In Group B, it was the task of the farmer to detect lame cows and to decide which cows required treatment and to initiate further measures if necessary. He did not know the results of the weekly locomotion scoring. The cows in Group B were treated either by the farmer himself or by the local veterinarian as usual. The number of first treatments in Group B was recorded.

### 2.4. Milk Yield, Fertility Measures, Health Data

Data from the monthly Dairy Herd Improvement (DHI) testing were collected from all lactating cows (milk kg). In addition, fertility measures were calculated from cows, which had their first insemination during the experimental period (interval from calving to first insemination, days open (= interval from calving to conception), first service conception rate (number of pregnant animals after first insemination × 100/ number of first inseminations)).

During the experimental period, 111 animals calved (n = 55 Group B; n = 56 Group A), 97 of which finished the puerperium (interval from calving until 42 days post partum). From these cows, the frequency of ovarian cysts, metritis/endometritis and clinical mastitis was recorded during regular herd health visits by the local veterinarian. In addition, all cows, which left the farm, were noted. 

### 2.5. Statistical Analysis

Cows were categorized into three score classes (locomotion score (LS) = 1, LS = 2, LS > 2) based on their individual locomotion scores. Statistical analysis was performed using SAS (Version 9.1, Statistical Analysis Institute, Cary, NC, USA) applying routine statistical procedures [15]. Data were checked for normal distribution (PROC UNIVARIATE). 

As normality could not be verified, non-parametric tests were performed for comparing the means (PROC NPAR1WAY wilcoxon): Interval from calving to first insemination, interval from calving to conception. 

Differences in frequencies between groups were compared with the Chi-square test (PROC FREQ): Locomotion score class, puerperal diseases, first service conception rate, cullings, frequencies of cows within groups with LS > 1 between weeks (Week 2 to Week 10, 20, 30, 40), lameness incidence over 4-week study intervals (number of new cases within 4 weeks × 100/number of non-lame cows at the beginning of each 4-week study interval, new case was defined as follows: Three times LS = 1 before the start of a 4-week study interval, at least one week with LS > 1 during these 4-week study intervals). For each new case of lameness (LS > 1 after at least three times LS = 1), lameness duration (weeks with LS > 1) was determined.

For 10 weeks (Week 2, 6, 10, 14, 18, 22, 26, 30, 34, 38) a logistic regression model was applied (PROC LOGISTIC) to predict the outcome of LS (LS = 1 *vs.* LS > 1 dependent variable) based on the variable groups (Group A *vs.* Group B), lactation number (LN) (LN = 1; LN = 2; LN > 2) and days in milk (DIM) (DIM ≤ 100 days, DIM ≤ 200 days, DIM > 200 days) as fixed effects and possible interactions. As there were no interactions, the interactions were excluded in the final model.

The 100-day milk yield was calculated using the first four DHI test day results applying Wood’s model [16] for cows, which had their first four DHI during the study period. Means were expressed as mean ± standard deviation (SD). For all tests, the level of significance was set at *P* < 0.05. At a *P*-value of ≥ 0.05 but < 0.1, differences were denoted as “trend”. 

## 3. Results

Table 2 shows an overview of the composition of the two experimental groups at the start of the study. In Group A (n = 99) and Group B (n = 99), 97 (98%) and 49 (49.5%) of the cows were treated at least once, respectively (*P* < 0.05).

**Table 2:** Summary statistics: Composition of groups (parity, days in milk (DIM), test-day milk yield) at the start of the study.

		Group A (total n = 99)	Group B (total n = 99)	*P*-value
Parity	frequency (n)			
	1	32.9 (27)	32.9 (28)	0.9364
	2	31.7 (26)	29.4 (25)	
	>2	35.4 (29)	37.7 (32)	
DIM	frequency (n)			
	≤100 days	25.0 (18)	29.2 (21)	0.5737
	>100 days	75.0 (54)	70.8 (51)	
Test-day milk yield				
	Mean ± SD (n)	25.99 ± 10.5 (67)	24.94 ± 9.7 (70)	0.4976
Number of animals				
	cows in milk	72	72	
	dry cows	10	13	
	heifers *	17	14	

* assigned after start of study.

### 3.1. Prevalence of Lameness Based on Locomotion Scores

**Figure 1:**
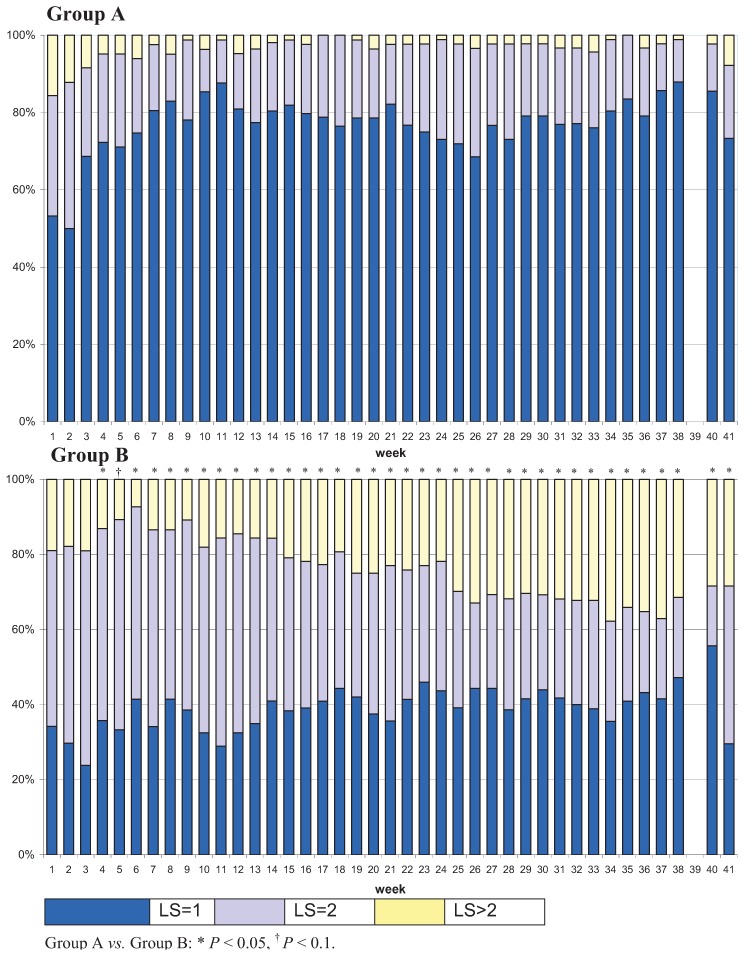
Distribution of cows in Group A and Group B among three locomotion score (LS) classes throughout a 41-week experimental period.

For the first three scoring dates, the weekly prevalence of LS = 1, LS = 2 and LS > 2 did not differ between Group A and Group B. Starting at Week 4, *i.e.*, only 3 weeks after claw trimming including all cows, the frequency of cows with LS = 1 was higher and that of LS = 2 and LS > 2 was lower in Group A compared with Group B (*P* < 0.05) (Figure 1). 

Over time, the percentage of cows with LS = 1 increased in Group A, whereas it decreased in Group B (Week 2 *vs.* Week 40: Group A: 50.00% *vs.* 85.56; Group B: 52.38% *vs.* 15.91%; *P* < 0.05). In the logistic regression the group effect was confirmed. From the 6th week onwards, cows from Group B were at a higher risk of having an LS > 1 compared with cows in Group A (*P* < 0.05). DIM had no significant effect (*P* ≥ 0.05). The lactation number was significant in each tested week. First lactating cows (LN < 2) had a lower risk for LS > 1 than pluriparous cows.

### 3.2. Duration of Lameness

The average duration of lameness for newly diseased animals was 3.7 ± 5.5 weeks in Group A (n = 112) and 10.4 ± 10.4 weeks in Group B (n = 135) (*P* < 0.001). 

### 3.3. Lameness Incidence

The four weekly lameness incidence was calculated from the second month of the experimental period onwards. Whereas it ranged from 8.0% to 22.6% in Group A, a wider range was noted in Group B (16.0% to 43.5%). Numerically, the four weekly incidence was always lower in Group A than in Group B. However, significant differences were only found in the 2nd, 4th and 5th month of the experimental period (*P* < 0.05). Within groups, the monthly lameness incidence did not differ (Table 3). 

**Table 3:** Four weekly incidence of new lameness cases.

Week	5-8	9-12	13-16	17-20	21-24	25-28	29-32	33-36
Group A	8.0	13.7	14.0	17.9	17.5	22.6	17.7	10.7
n	2/25	7/51	8/57	10/56	10/57	12/53	9/51	6/56
Group B	43.5	16.0	41.9	39.1	23.5	30.0	36.8	20.0
n	10/23	4/25	13/31	9/23	4/17	6/20	7/19	3/15
Total	25.0	14.5	23.9	24.1	18.9	24.7	22.9	12.7
n	12/48	11/76	21/88	19/79	14/74	18/73	16/70	9/71
*P*-value	0.046	0.791	0.003	0.045	0.580	0.515	0.089	0.337

### 3.4. Reproductive Measures

In the experimental period, 123 animals were inseminated for the first time following calving and were, therefore, evaluated with regards to reproductive measures. The mean interval from calving to first insemination or conception and the first service conception rate did not differ between Group A and Group B (*P* ≥ 0.05) (Table 4). 

**Table 4:** Reproductive measures of cows in Group A and Group B.

	Group A (n)	Group B (n)	*P*-value
Interval calving to 1st insemination (d) Mean ± SD	76.2 ± 18.9 (62)	74.6 ± 23.4 (61)	0.559
Days open (d) Mean ± SD	147.5 ± 23.4 (58)	138.4 ± 83.8 (59)	0.321
First service conception rate (%)	27.4 (62)	32.8 (61)	0.516

### 3.5. Diseases during the Puerperium

In total, 97 animals calved during the experimental period and finished their puerperium (Group A: n = 50; Group B: n = 47). The incidence of clinical mastitis, metritis/endometritis and ovarian cysts did not differ between groups. In Group A, there were 24.0% disease cases compared with 34.0% cases in Group B (*P* = 0.275) (Table 5). 

**Table 5:** Incidence (%) of ovarian cysts, metritis/endometritis and mastitis during the puerperium (until 42 d *post partum*) in Group A and Group B.

	Without disease % (n)	Ovarian cysts % (n)	Metritis/endometritis % (n)	Mastitis % (n)
Group A (50)	76.0 (38)	6.0 (3)	16.0 (8)	2.0 (1)
Group B (47)	66.0 (31)	6.4 (3)	25.5 (12)	2.1 (1)
*P*-value	0.275	0.811	0.232	0.887

### 3.6. Milk Yield

The mean 100-d milk yield was 3,386 ± 174.68 kg in Group A (n = 75) compared with 3,359 ± 169.46 kg in Group B (n = 76) indicating a difference between groups (*P* = 0.084). 

## 4. Discussion

The prevalence of lame cows (LS > 1) at the beginning of this study was found to be 50% in Group A and 47.6% in Group B, when locomotion scores >1 were considered as lame. The weekly lameness control and consistent treatment of lame cows (LS ≥ 2) reduced the prevalence in Group A significantly (14.4% in week 40). There are a number of studies on the prevalence and incidence of lameness in dairy cows [17,18,19] with prevalence estimates ranging from 5% to 45%. This wide variation is partly due to the different scoring systems used. In studies using similar scoring systems as well as lameness definitions, the mean prevalence ranged from 22.6% to 33.2% [8,9].

In the current study, the overall monthly incidence ranged from 12.7% to 25.0%, and did not change significantly over time in either group. Clarkson *et al.* [20] collected data from 37 dairy farms in Great Britain. The mean annual incidence was 54.6 new cases per 100 cows with a range from 10.7 to 170.1. Alawneh *et al.* [21] found an overall incidence risk of 13%.

Early detection and treatment of lame cows reduced lameness prevalence in the study cohort. However, there was no effect on lameness incidence within each group. As the prevalence is a function of incidence and duration of illness, the reduction of prevalence could be attributed to a decrease in the duration of lameness. In fact, the duration of lameness was almost three times longer in Group B than in Group A. This is in accordance with a hypothesis by Green *et al.* [22] suggesting that rapid detection and treatment reduce the duration of lameness and hence are beneficial from a welfare point of view, but have no impact on the incidence in general. Clarkson *et al.* [20] conducted a survey on the incidence and prevalence of lameness in adult dairy cattle on 37 farms in four regions of Great Britain. A comparison of the mean annual incidence and mean annual prevalence of lameness on the 37 farms revealed that they were correlated significantly. The comparison also disclosed that there was a high incidence of lameness on the Somerset farms, but a low prevalence. As explanation, the authors presumed that the duration of the lameness incidents was very short in this region, because the farmers in Somerset had been trained to report cases very early and this early reporting was followed by veterinary treatment. Clarkson *et al.* [20] support the view that the training of farmers to recognize lameness and to ensure veterinary treatment of lame cows would reduce the duration of lameness and thus improve the welfare of lame cows. In accordance with our results, they also found that this approach would not necessarily reduce the incidence of new cases, since the incidence depended on other risk factors.

While the incidence did not decrease within each group, differences in incidence were noted, reaching significance in three of the eight study intervals. To our knowledge, there is no general connection between early treatment and incidence. Lameness can have a number of different etiologies, and while some of them might be eliminated through early and more frequent treatment (e.g., dermatitis digitalis, laminitis), most of them are multifactorial and not responsive to treatment only (without taking other risk factors like husbandry conditions, nutrition, and herd management into account). Hence, we do not believe that treatment alone has an impact on the incidence of lameness in our study and rather that the differences between Group A and B are coincidental, especially since the difference could not be shown for all study intervals. 

Leach *et al.* [12] also showed that early treatment reduced the prevalence of lameness 4 weeks after treatment. In their study the early threshold schedule resulted in a much shorter time to treatment than the conventional approach. In contrast to our study, the experimental design of the study of Leach *et al* excluded cows with repeated cases of lameness. Therefore, in their study the difference in prevalence of lame cows between groups declined with increasing time. It needs to be mentioned that while the prevalence in Group A decreased, prevalence in Group B increased from the baseline level during the study period. This is most likely attributable to the fact that the dairy farm participated in a herd health management program of this particular farm prior to the study. While this program mainly focused on reproductive issues, the herd health veterinarians pointed out lame animals to the farmer and treated them during their visits. As soon as the study started, animals in Group B were only treated when the farmer specifically requested it or did so himself or consulted another veterinarian. Hence it can be assumed that when left to his own judgment, the farmer chose to treat fewer animals than would have been the case during the herd health management visits, which explains the increase in prevalence of lameness in Group B. Another reason could be that the putative positive effects of claw trimming might have declined over time in Group B, whereas in Group A almost every animal was subjected to lameness treatment at least once.

Even though the mean lameness score was significantly lower in Group A from Week 4 onwards, a positive effect of early detection and treatment of lameness on the reproductive performance and incidence of ovarian cysts, mastitis or endometritis could not be shown. However, there was a trend towards higher 100-d milk yield in Group A when compared to Group B. This is consistent with the findings of other studies showing a reduced milk yield in lame dairy cows [1,22,23] with an estimated milk loss per cow of 1.5–2.8 kg per day for the first 2 weeks after diagnosis [1,6] up to 2 kg per day for up to five months before and after diagnosis in the UK [2].

Although the results for lameness prevalence were promising, we cannot rule out the possibility of factors other than early diagnosis and treatment, such as higher treatment frequencies and consistent treatment quality, influencing the prevalence in the present study. Other limitations of the study are the small sample size, the short duration of the study and the focus on one study farm only, which might also explain the lack of positive effects on health and reproductive performances.

## 5. Conclusion

Lameness in dairy cows is one of the main welfare issues. Reducing the prevalence of lameness should be one of the most important goals in dairy farming. The study outcomes suggest that early detection and treatment of lame cows (including slightly lame cows) increase the number of cows having to undergo lameness treatment and reduce the duration of lameness, with a positive effect on prevalence of lameness.

Further studies are needed to test the efficiency of this lameness management program on a higher number of farms especially under the aspect of practicability. In addition, other measures to reduce the incidence of lameness should be considered. 

## References

[1] Warnick L.D., Janssen D., Guard C.L., Grohn Y.T. (2001). The effect of lameness on milk production in dairy cows. J. Dairy Sci..

[2] Green L.E., Hedges V.J., Schukken Y.H., Blowey R.W., Packington A.J. (2002). The impact of clinical lameness on the milk yield of dairy cows. J. Dairy Sci..

[3] Hernandez J., Shearer J.K., Webb D.W. (2002). Effect of lameness on milk yield in dairy cows. J. Am. Vet. Med. Assoc..

[4] Collick D.W., Ward W.R., Dobson H. (1989). Association between types of lameness and fertility. Vet. Rec..

[5] Hernandez J., Shearer J.K., Webb D.W. (2001). Effect of lameness on the calving-to-conception interval in dairy cows. J. Am. Vet. Med. Assoc..

[6] Rajala-Schultz P.J., Grohn Y.T. (1999). Culling of dairy cows. Part I. Effects of diseases on culling in Finnish Ayrshire cows. Prev. Vet. Med..

[7] Galindo F., Broom D.M. (2002). Effects of lameness of dairy cows. J. Appl. Anim. Welf. Sci..

[8] Tadich N., Flor E., Green L. (2010). Associations between hoof lesions and locomotion score in 1098 unsound dairy cows. Vet. J..

[9] Sogstad A.M., Fjeldaas T., Osteras O. (2012). Locomotion score and claw disorders in Norwegian dairy cows, assessed by claw trimmers. Livest. Sci..

[10] Whay H.R., Main D.C.J., Green L.E., Webster A.J.F. Farmer perception of lameness prevalence. Proceedings of the 12th International Symposium on Lameness in Ruminants.

[11] Leach K.A., Whay H.R., Maggs C.M., Barker Z.E., Paul E.S., Bell A.K., Main D.C.J. (2010). Working towards a reduction in cattle lameness: 1. Understanding barriers to lameness control on dairy farms. Res. Vet. Sci..

[12] Leach K.A., Tisdall D.A., Bell N.J., Main D.C.J., Green L.E. (2012). The effects of early treatment for hindlimb lameness in dairy cows on four commercial UK farms. Vet. J..

[13] Archer S., Bell N., Huxley J. (2010). Lameness in UK dairy cows: A review of the current status. In Pract..

[14] Sprecher D.J., Hostetler D.E., Kaneene J.B. (1997). A lameness scoring system that uses posture and gait to predict dairy cattle reproductive performance. Theriogenology.

[15] Steel R.G.D., Torrie J.H. (1980). Principles and Procedures of Statistics: A Biometrical Approach.

[16] Wood P.D.P. (1977). Biometry of lactation. J. Agric. Sci..

[17] Manske T., Hultgren J., Bergsten C. (2002). Prevalence and interrelationships of hoof lesions and lameness in Swedish dairy cows. Prev. Vet. Med..

[18] Winckler C., Brill G. Lameness prevalence and behavioural traits in cubicle housed dairy herds—A field study. Proceeding of the 13th International Symposium and 5th Conference on Lameness in Ruminants.

[19] Espejo L.A., Endres M.I., Salfer J.A. (2006). Prevalence of lameness in high-producing Holstein cows housed in freestall barns in Minnesota. J. Dairy Sci..

[20] Clarkson M.J., Downham D.Y., Faull W.B., Hughes J.W., Manson F.J., Merritt J.B., Murray R.D., Russell W.B., Sutherst J.E., Ward W.R. (1996). Incidence and prevalence of lameness in dairy cattle. Vet. Rec..

[21] Alawneh J.I., Stevenson M.A., Williamson N.B., Lopez-Villalobos N., Otley T. (2012). The effect of clinical lameness on liveweight in a seasonally calving, pasture-fed dairy herd. J. Dairy Sci..

[22] Green L.E., Borkert J., Monti G., Tadich N. (2010). Associations between lesion-specific lameness and the milk yield of 1,635 dairy cows from seven herds in the Xth region of Chile and implications for management of lame dairy cows worldwide. Anim. Welf..

[23] Reader J.D., Green M.J., Kaler J., Mason S.A., Green L.E. (2011). Effect of mobility score on milk yield and activity in dairy cattle. J. Dairy Sci..

